# The role of skin-to-skin contact in exclusive breastfeeding: a cohort study

**DOI:** 10.11606/s1518-8787.2022056004063

**Published:** 2022-07-21

**Authors:** Marivanda Julia Furtado Goudard, Zeni Carvalho Lamy, Sérgio Tadeu Martins Marba, Geisy Maria de Souza Lima, Alcione Miranda dos Santos, Marynea Silva do Vale, Talyta Garcia da Silva Ribeiro, Roberta Costa, Vivian Mara Gonçalves de Oliveira Azevedo, Fernando Lamy-Filho

**Affiliations:** I Universidade Federal do Maranhão Departamento de Saúde Pública Programa de Pós-Graduação em Saúde Coletiva São Luís MA Brasil Universidade Federal do Maranhão . Departamento de Saúde Pública . Programa de Pós-Graduação em Saúde Coletiva . São Luís , MA , Brasil; II Universidade Estadual de Campinas Departamento de Pediatria Faculdade de Ciências Médicas Campinas SP Brasil Universidade Estadual de Campinas . Departamento de Pediatria da Faculdade de Ciências Médicas . Campinas , SP , Brasil; III Instituto de Medicina Integral Professor Fernando Figueira Departamento de Neonatologia Recife PE Brasil Instituto de Medicina Integral Professor Fernando Figueira . Departamento de Neonatologia . Recife , PE , Brasil; IV Hospital Universitário Universidade Federal do Maranhão Departamento de Neonatologia São Luís MA Brasil Hospital Universitário da Universidade Federal do Maranhão . Departamento de Neonatologia . São Luís , MA , Brasil; V Universidade Federal do Maranhão Faculdade de Medicina São Luís MA Brasil Universidade Federal do Maranhão . Faculdade de Medicina . São Luís , MA , Brasil; VI Universidade Federal de Santa Catarina Departamento de Enfermagem Florianópolis SC Brasil Universidade Federal de Santa Catarina . Departamento de Enfermagem . Florianópolis , SC , Brasil; VII Universidade Federal de Uberlândia Faculdade de Educação Física e Fisioterapia Uberlândia MG Brasil Universidade Federal de Uberlândia . Faculdade de Educação Física e Fisioterapia . Uberlândia , MG , Brasil

**Keywords:** Infant, Low Birth Weight, Premature Birth, Breast Feeding, Kangaroo-Mother Care Method, Intensive Care, Neonatal

## Abstract

**OBJETIVE:**

To understand the role of exposure to skin-to-skin contact and its minimum duration in determining exclusive breastfeeding at hospital discharge in infants weighing up to 1,800g at birth.

**METHODS:**

A multicenter cohort study was carried out in five Brazilian neonatal units. Infants weighing ≤ 1,800g at birth were eligible. Skin-to-skin contact time was recorded by the health care team and parents on an individual chart. Maternal and infant data was obtained from maternal questionnaires and medical records. The Classification Tree, a machine learning method, was used for data analysis; the tree growth algorithm, using statistical tests, partitions the dataset into mutually exclusive subsets that best describe the response variable and calculates appropriate cut-off points for continuous variables, thus generating an efficient explanatory model for the outcome under study.

**RESULTS:**

A total of 388 infants participated in the study, with a median of 31.6 (IQR = 29–31.8) weeks of gestation age and birth weight of 1,429g (IQR = 1,202–1,610). The exclusive breastfeeding rate at discharge was 61.6%. For infant’s weighting between 1,125g and 1,655g, exposed to skin-to-skin contact was strongly associated with exclusive breastfeeding. Moreover, infants who made an average > 149.6 min/day of skin-to-skin contact had higher chances in this outcome (74% *versus* 46%). In this group, those who received a severity score (SNAPPE-II) equal to zero increased their chances of breastfeeding (83% *versus* 63%).

**CONCLUSION:**

Skin-to-skin contact proved to be of great relevance in maintaining exclusive breastfeeding at hospital discharge for preterm infants weighing 1,125g–1,655g at birth, especially in those with lower severity scores.

## INTRODUCTION

Premature birth can compromise the breastfeeding process due to the biological immaturity of the preterm infant and the mother-child separation due to the need for neonatal hospitalization ^1 , 2^ . This separation can also harm interaction relationships that would be built in this sensitive and critical period after birth, when the first suckling at the breast should occur ^3^ .

Seeking to minimize the negative effects of mother-child separation, early skin-to-skin contact (SSC) is recommended during hospitalization in the neonatal unit, which consists of placing the infant in an upright position, wearing only diapers, on the mother’s chest ^4^ . Contact with the mother, her touch and speech promote breastfeeding ^5 , 6^ , stimulate lactation, and the progression of the sucking behavior in both full-term ^3 , 4 , 6^ and preterm infants ^7^ .

The World Health Organization recommends the use of SSC as a routine to care for infants ^4^ , since evidence shows that this practice promotes breastfeeding, reduces hypothermia, neonatal mortality, sepsis, and hospital stay length in preterm infants and/or newborns with low birth weight ^11 , 12^ . Furthermore, SSC is associated with greater emotional regulation, and better cognitive and motor development in the first year of life for these infants ^13^ .

However, despite the association between SSC and exclusive breastfeeding (EBF) at hospital discharge being well established in the literature, how this kind of care fits into a broader explanatory framework, composed of other important elements in determining this outcome, is unclear. The literature also shows conflicting results regarding the length of SSC needed for the positive effects on breastfeeding ^9 , 14 , 15^ . Two meta-analysis studies investigated cut-off points for SSC time in neonatal outcomes and presented the failure to determine the minimum time needed to observe these associations as a limitation ^11 , 12^ .

Thus, our study aimed to understand the role of skin-to-skin contact in determining EBF at hospital discharge in infants weighing up to 1800 g at birth and investigated the minimum time required for this possible protective effect to appear. The answer to this question is extremely important to contribute to the health policies that support breastfeeding, providing a better quality of life for these infants, especially in low- and middle-income countries.

## METHODS

### Study Design, Setting, and Sample

This prospective cohort study is part of a multicenter research entitled “Effect of exposure time to SSC on clinical outcomes in low-birth-weight infants”. Five Brazilian neonatal units – considered references in Brazil for the Kangaroo Method – participated in this research, two from the Northeast, two from the Southeast, and one from the South of the country. The study was conducted from May 2018 to March 2020 after approval by ethics committee – Certificate of Presentation of Ethical Appreciation (CAAE Nº. 83803817.0.1001.5086). All parents who participated in the research signed the informed consent form.

**Figure 1:**
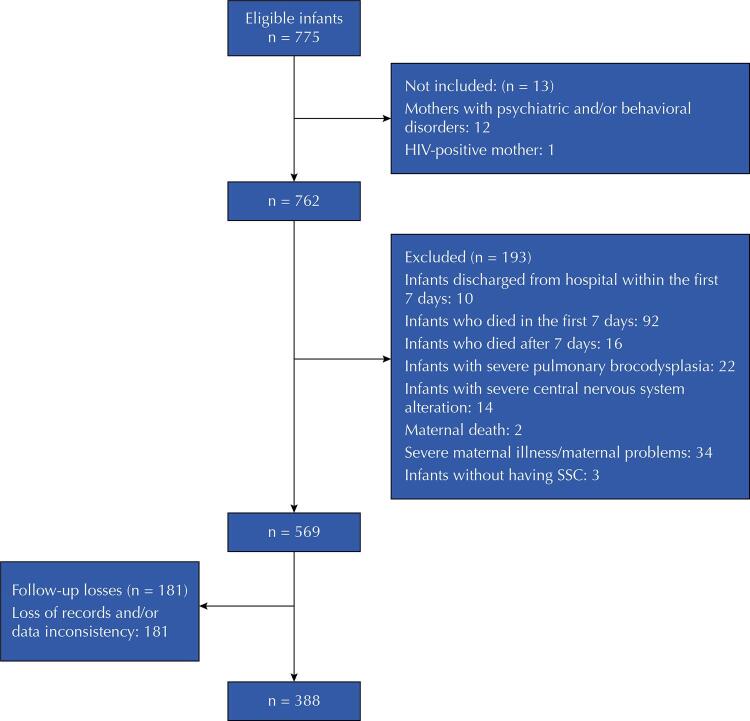
Flowchart of sample selection.

HIV: human immunodeficiency virus; SSC: skin-to-skin contact.

All live births in these institutions during the study period that met the following criteria were considered eligible: single delivery, birth weight up to 1,800g, and no malformations, severe perinatal asphyxia and/or genetic syndromes. The sample flowchart shows non-inclusion and exclusion criteria, as well as losses ( Figure 1 ).

The definition of a cut-off point of ≤ 1,800g considered a pilot study that showed that infants with higher weights spent less time in the neonatal units, which could interfere with the observation of the effect of SSC on the outcome under study.

For this research, the minimum sample size was set at 357 dyads. This was based on a pilot study that considered a 0.6 exposed/unexposed ratio and a 23% risk difference. A bilateral significance level of 95%, power of 99%, and type I error of 5% were considered.

### Data Collection

Maternal and infant variables were collected by using questionnaires applied to mothers, and completed with data from medical records, during the period of hospitalization of the neonate. The SSC was registered on cards attached to the bedside and filled out by the health team during the infant’s hospital stay. Parents were trained and encouraged to also register under the supervision of the health team. When the infant went to the SSC, the time the contact started, the time it ended, and who performed it (father or mother) were recorded. This procedure was performed at each new SSC during hospitalization.

The SSC records were checked daily by previously trained research assistants, who encouraged parents and health professionals to continue the records and consolidated the total daily SSC time in a specific form.

### Variables

Variables related to maternal and infant characteristics and SSC time were collected to be included in the theoretical model, built from literature data, as follows:

Main outcome: EBF *at hospital discharge* (when this form of feeding was reported in the medical record on the day of hospital discharge).

Exposure variable: *time of exposure to SSC per day* , calculated by dividing the total time of SSC practiced during hospitalization, in minutes, by the number of days on which this contact was made.

Explanatory variables relative to the mothers: Age (less than 20 years, from 20 to 34, and above 35 years); Education level (no education/incomplete elementary, complete elementary/incomplete high school, complete high school/incomplete high school, and complete high school); Marital status (with partner or without partner); Specific hypertensive gestation syndrome (SGHS); Use of alcohol during pregnancy (yes or no); Infection during pregnancy (yes or no); Type of delivery (vaginal or cesarean); Use of corticosteroids before delivery (yes or no); Adequacy of prenatal care (adequate or no prenatal care/inadequate), considered adequate the prenatal care beginning until the 4th month of gestation and six or more consultations performed for a full-term pregnancy or a smaller number according to the gestational age at delivery (three consultations until 29 weeks; four consultations from 30 to 33 weeks and five consultations from 34 to 36 weeks).

Explanatory variables relative to the infants: Birth weight in grams; Apgar score at the 5th minute of life; Gestational age (GA) at birth in weeks, calculated by the date of last menstrual period or first trimester ultrasound or the New Ballard score; Adequacy of weight for gestational age, categorized as adequate, small, or large for gestational age, according to Intergrowth 21 classification ^16^ ; Score for Neonatal Acute Physiology – Perinatal Extension II (SNAPPE II), scored from 0 to 162 ^17^ ; Early infection (within the first 48 hours of life of the infant); Late infection (> 48 hours of life of the infant); Time to perform the first SSC during hospitalization (in days).

The collected data was tabulated in a Google Form ^®^ instrument and then exported to a Microsoft Office Excel ^®^ , version 2016.

### Statistical Analysis

Quantitative data were represented by mean and standard deviation or medians and interquartile ranges, depending on distribution and normality criteria and categorical variables were presented as frequencies and percentages. To calculate the statistical difference between the variables GA and birthweight of the study sample and losses to follow-up, the Mann-Whitney test was used, which compares two independent means when impossible to assume the normal distribution of these variables.

For the statistical analysis, the Classification Tree (CT) was used, a non-parametric method based on machine learning (artificial intelligence) capable of building data prediction models. The CT created an algorithm to identify the most important variables in the data set of the theoretical model and to develop an efficient explanatory model for the outcome under study ^18^ . The dependent variable used was “EBF at discharge”, considered the “root node”, and, from the set of predictor variables, the descendant nodes were selected and identified, by logical tests, until these nodes stopped dividing, ending this growth in “terminal nodes”. When analyzing continuous variables, the CT algorithm itself determined the most statistically appropriate cut-off points. Chi-Square Automatic Interaction Detection (CHAID) method was used to grow the tree ^19^ . The method maximized the significance of the chi-square statistic in each partition, which characterized CHAID as a structure of tests of significance. Due to the successive comparison tests applied in this technique, a correction factor for Bonferroni inequality was calculated by the CT algorithm, to obtain an adjusted level of significance. Thus, evaluating the entry of each variable in the model and verifying whether its contribution was significant or not among the predictor variables was possible. In summary, the CT, partitioned the dataset into mutually exclusive subsets that best described the response variable, exhaustively, using machine learning for this. The statistical package Stata ^®^ 14.0 was used for the descriptive analysis and the IBM SPS Statistic ^®^ for the CT.

## RESULTS

We analyzed 388 infants ( Figure 1 ), with a median gestational age of 31.6 weeks and a weight of 1,429g. The EBF rate at discharge was 61.6%. Table 1 shows other characteristics of the infants. Table 2 shows maternal characteristics.

**Table 1:** Characterization of infants ≤ 1,800g (n = 388) admitted from five Brazilian centers.

Variables	n (%)	m (IQR)
Male	198 (51.0)	-
AGA (adequate for GA)	245 (63.1)	-
SGA (Small for GA)	134 (34.5)	-
Early infection	92 (23.7)	-
Late infection	102 (26.3)	-
EBF at discharge	239 (61.6)	-
Partial breastfeeding at discharge	126 (32.5)	-
Exclusive infant formula at discharge	23 (5.9)	-
Gestational age (weeks)	-	31.57 (31.8–29.0)
Weight (grams)	-	1,429 (1,610–1,202.5)
SSC/day (minutes)	-	150.23 (275–108.3)
Apgar 5 ^th^ minute	-	9 (9–8)
SNAPPE II	-	5 (13–0)
Time to start 1 ^st^ SSC (in days)	-	5 (8–4)
Time to start 1 ^st^ enteral feed (in days)	-	1 (2–1)
Days hospitalized	-	34 (52.5–25)

GA: gestational age; SSC: skin-to-skin contact; EBF: exclusive breastfeeding; SNAPPE II : score for neonatal acute physiology – perinatal extension II. M (IQR): mean and interquartile range.

**Table 2:** Demographic, socioeconomic, and gestational characteristics of mothers of infants ≤ 1,800g (n = 388), from five Brazilian centers.

Variables	n (%)
Age (years)	
< 20	63 (16.24)
20–34	244 (62.89)
≥ 35	81 (20.88)
Education	
No education/incomplete elementary school	43 (11.08)
Complete elementary school/incomplete high school	104 (26.8)
Complete high school/incomplete higher education	193 (49.74)
Complete higher education	43 (11.08)
Ignored	5 (1.29)
Marital status	
With partner	317 (82.12)
Pre-natal care	
Adequate	302 (77.84)
Used alcohol during pregnancy	34 (8.76)
Used corticoids	
Yes	293 (75.52)
Specific hypertensive gestation syndrome	
Yes	203 (52.32)
Infection during pregnancy	
Yes	151 (38.92)
Type of birth	
Cesarian	263 (67.78)

SNAPPE II: score for neonatal acute physiology – perinatal extension II.

We lost follow-up of 181 of the infants (23.9%) ( Figure 1 ). However, these infants showed no significant difference compared with the population included in the study, in terms of GA (30.9 ± 3.2 *versus* 31.4 ± 2.6 weeks; p = 0.068) and birth weight (1,345.6g ± 335.7g *versus* 1,388.6g ± 285.3g; p = 0.302).

**Figure 2:**
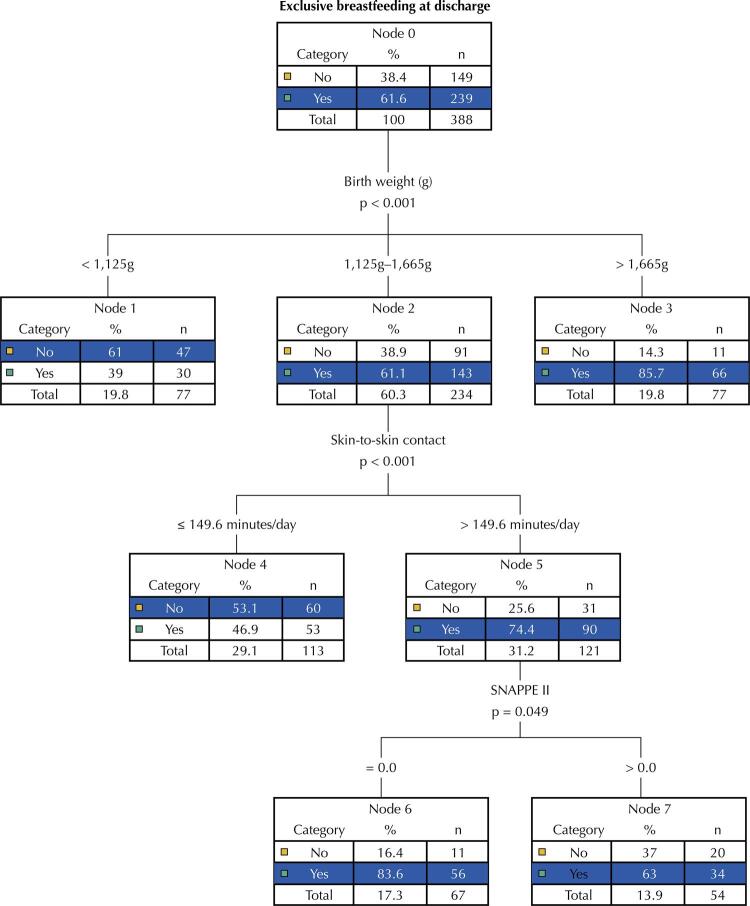
Exclusive breastfeeding classification tree at hospital discharge, according to maternal and infant characteristics from five Brazilian centers.

Figure 2 shows the CT with the outcome EBF adjusted to explanatory variables relative to the mothers and infants. The tree exposes the relationship between the characteristics of the dyads in three levels of depth and none of the maternal characteristics remained associated with the outcome in the explanatory model.

The variables and their most relevant cut-off points were birth weight (between 1,125g – 1,665g), SSC time (> 149.6 min/day), and SNAPPE-II severity score (= 0). The first variable associated with EBF at the classification tree was birth weight. Infants over 1,665g at birth had an 85.7% chance of EBF at discharge. In those with a weight range between 1,125g and 1,655g who underwent SSC for more than 149.6 minutes/day, the chance of EBF at discharge increased from 61.1% to 74.4% compared with the group with shorter times of practice. The group that had longer SSC time and zero severity score at admission also obtained an 83.6% probability for EBF ( Figure 2 ).

## DISCUSSION

The SSC duration greater than 149.6 minutes/day (around 2 hours and 30 minutes) showed a strong association with EBF at hospital discharge in newborns with birth weight between 1,125g and 1,655g. Also, those who performed longer SSC/day and who scored zero SNAPPE-II at admission had a greater chance of this outcome (83.6% *versus* 63%).

The practice of SSC has been associated with shorter time to reach full enteral diet ^20^ , earlier start of breastfeeding ^21^ , longer duration of first suckling at the breast ^3^ , and higher rates of EBF at hospital discharge when compared with conventional care ^8 , 11 , 12 , 22^ .

We established the role of the SSC in determining the chances of EBF at the time of hospital discharge for infants admitted to the neonatal intensive care unit (NICU) by constructing the CT, which considered the contribution of several maternal and neonatal variables used in the theoretical model of this study.

Birth weight was the variable that best explained the outcome (EBF at discharge), being the most relevant to determine it. The CT algorithm subdivided the variable into three weight categories: < 1,125g (node 1), 1,125g – 1,665g (node 2), and > 1,665g (node 3). Nodes 1 and 3 are terminal, while node 2 was influenced by two other variables that increase the chances of EBF at discharge.

Node 2 represents more than 60% of the studied sample, which comprises an intermediate birthweight category (1,125g – 1,665g) and the chance of EBF at discharge among these infants was 61.1%. At this point, the CT creates a new subdivision introducing the SSC exposure time as a new explanatory and modifying factor for this probability. The algorithm establishes a cut-off point of 149.6 minutes (approximately two and a half hours), which created two new categories (nodes 4 and 5). Those who practiced more SSC/day showed a positive effect compared with those with less time. We observed the highest possibility of discharge with EBF (74%) in those who underwent SSC for more than 149.6 min/day (node 5), which represents an increase of 13.3 percentage points in the EBF rate for this intermediate weight range.

This denotes a clear influence of the time of SSC in the results of breastfeeding in this birth weight range. These results are even more relevant considering this weight range tends to be the most prevalent among preterm infants admitted to the NICU. This probability of EBF at discharge was also higher than the highest rate found (51.5%) in countries in various regions of Europe, in very preterm infants, where the practice of SSC varies greatly ^23^ .

The literature confirms the association between longer SSC times and greater chances of EBF at discharge. A similar study in Sweden found an association between preterm infants that had over 139 min/day of SSC with any form of breastfeeding (exclusive or supplemented with a formula) until the first month of corrected age ^24^ . The SSC cut-off point of 149.6 min/day observed in our study was specifically associated with EBF (the gold standard of infant nutrition) ^4 , 14^ , which can directly contribute to their greater survival and better neurodevelopment after hospital discharge.

In the explanatory graphic model of EBF determinants at discharge from the NICU, CT creates yet another subdivision among those infants with a longer SSC time (> 149.6 min), which is another factor that can influence this outcome: the SNAPPE-II score (infant severity index at admission). For the creation of these nodes (6 and 7), the cut-off point found by the CT algorithm was SNAPPE-II = 0. This means that the chances of breastfeeding increased from 74.4% in the previous node to 86.6% for those infants who received a score of zero. SNAPPE-II is a useful tool to assess the severity of infant’s illness and its correlation with neonatal mortality ^17^ . Thus, we expected that infants with lower severity rates would be more likely to maintain breastfeeding. However, regardless of severity score, infants who performed longer SSC received a significant positive effect on EBF at discharge (increase to 74.4% compared to the previous node of 61.1%).

Nodes 1 and 3 of CT represent weight ranges that comprise, in a greater proportion, extreme-preterm infants (< 1,125g) and late-preterm infants (> 1,655g), respectively ^16^ . Each of these nodes also represents only 19.8% of the study sample. We observed the highest chance of discharge with EBF (85.7%) in the birth weight range greater than 1,655 g (node 3), which agrees with the literature. Late-preterm infants (34–36 weeks) are subject to less severe morbidities, such as mild breathing difficulties, hyperbilirubinemia, and hypoglycemia ^10^ , which increases the chances of positive results, such as maintaining EBF at discharge.

A recent meta-analysis with a sample composed of late-preterm infants with an average weight of 2,312g (in the group using SSC) and 2,300g (in the control group) observed no statistically significant difference in breastfeeding rates at hospital discharge ^15^ . Late-preterm infants are not subject to the same challenges as very preterm infants, such as longer hospital stays and greater difficulties in initiating and maintaining breast suction coordination ^1 , 2^ .

Node 1 (weight range < 1,125g) is at the other end of these categories. Infants in this category had a chance of EBF at discharge of only 39%. This weight range is correlated with extreme prematurity ^16^ , which can lead to additional complications such as intraventricular hemorrhage, periventricular leukomalacia, necrotizing enterocolitis, retinopathy of prematurity, among others, and can lead to the future development of neurobehavioral disorders ^25^ , thus reducing chances of maintaining EBF at discharge. However, the practice of SSC should be encouraged for this weight range since it can contribute to any form of breastfeeding at discharge for these extreme preterm infants, as a study of infants with less than 29 weeks of gestational age observed ^9^ .

In summary, the CT outlines a scenario in which the chances of EBF are maximized in two situations: infants weighing more than 1,655g at birth (represented by a small proportion of the sample) and infants with birth weight between 1,125g and 1,655g (higher proportion of hospitalized infants in this study) and who underwent more than 149.6 min/day of SSC. In the latter situation, the probability of EBF also increased for those with a null severity score at admission.

The EBF contributes to a better neuro-psychomotor development and better quality of life for these infants, especially if extended to the sixth month of corrected age or as long as possible ^4 , 13 , 26^ . The scenario outlined in this study denotes that the use of SSC, especially if the daily average is greater than 149.6 minutes, is extremely relevant and can maximize the chances of EBF at discharge in most preterm infants admitted to the NICU.

As a limitation of the study, the absence of follow-up after hospital discharge stands out, at which time the maintenance of EBF becomes more challenging and tends to decrease over the months. Research in this regard is still needed. Another limitation was the loss of follow-up of 23.9% of the infants. However, this group of infants showed no statistical difference when compared with the sample group studied regarding the variables GA and birth weight.

The participation of units from institutions in three different regions of the country is a positive point of the work. The prospective data collection and the choice of a method that allowed us to observe the cut-off point of the duration of this practice associated with the outcome in question are strengths of the study. We could create an explanatory model for the practice of EBF at the time of discharge from the neonatal unit, adjusted by several explanatory variables, and situate the practice of SSC in this context.

## CONCLUSIONS

This study reinforces SSC as a good neonatal care practice that contributes to increasing the chances of positive results in infants up to 1,800g at birth who need intensive care. We recommend a contact duration greater than 149.6 minutes/day to maximize the chances of a positive effect on EBF, especially among infants with birth weight of 1,125g to 1,655g admitted to the neonatal unit.
